# The impact of education on subjective well-being: a meta-analysis based on 59 empirical studies

**DOI:** 10.3389/fpsyg.2025.1651896

**Published:** 2025-08-21

**Authors:** Xuemin Dong, Zhe Xing, Haixin Song, Dexin Hu

**Affiliations:** ^1^School of Education, Beijing Institute of Technology, Beijing, China; ^2^School of Education, Tianjin University, Tianjin, China

**Keywords:** educational level, higher education participation, lifelong learning engagement, subjective well-being, meta-analysis, moderating factors

## Abstract

**Introduction:**

Subjective well-being (SWB) has emerged as a prominent research focus, especially in the context of specific dimensions of education (educational level, higher education participation, and lifelong learning engagement). This study aimed to assess whether education (educational level, higher education participation, lifelong learning engagement) influences SWB, and to explore whether moderators such as rural -urban residence and publication year alter this relationship.

**Methods:**

Following the PRISMA guidelines, we conducted a systematic review and meta-analysis. After applying rigorous eligibility criteria (e.g., empirical studies reporting relationships between education and SWB, specific published years from 2012 to 2023 and languages including English and Chinese), this study screened 59 empirical studies and extracted 185 effect sizes that could be used in the meta-analysis. First, we examined the relationship between education and SWB and further analyzed the moderators to explore the effects of rural -urban factors and publication year.

**Results:**

(1) Educational level and higher education participation significantly influenced SWB, while lifelong learning engagement showed a weaker but significant positive association, partially supporting our initial hypothesis. (2) The relationship between education and SWB was moderated by rural -urban factors. (3) Publication year within the studied time also exerted a significant moderating effect.

**Discussion:**

This study clarified that education should be emphasized continuously, and education equity ought to be improved further so that both rural and urban residents gain high levels of SWB in the future. These results of the paper will provide insights into how SWB interacts with education and offer useful suggestions of improving SWB from an educational perspective.

## 1 Introduction

Education is a primary source of human capital that is strongly connected to the well-being of modern societies. Hundreds of academic studies show that more educated people usually have more secure and high-benefit job opportunities, have greater labor force flexibility, live longer and healthier, are less likely to be affected by unemployment trends, and ultimately receive not only income levels represented by high salaries but also non-monetary benefits such as lifetime subjective well-being (SWB) (Green, 2011). However, a growing body of literature has documented a negative and insignificant correlation between education and SWB. One possible explanation for these puzzling results is that despite education endows benefits, graduates may face significant stress such as competitive job markets, high expectations, and potential work-life imbalance, leading to heightened anxiety (Woolston, 2022), which might act as a counteracting force, partially offsetting the positive well-being gains from education and contributing to a weakened or non-significant overall association. Another possible explanation is that an increasing number of studies use reduced SWB regressions forms that often control for variables such as age, gender, health, income, and marital status, and thus close channels, but these variables are actually key intermediaries through which education typically boosts SWB (Jebb et al., 2020; Voukelatou et al., 2021). By statistically accounting for these downstream outcomes, such analyses unintentionally “block the paths” connecting education to SWB. This hides the real underlying positive relationship, often leading to findings that aren’t statistically significant. Hence, what is the relationship between education and SWB? What factors account for the variations in the estimates of SWB from the perspective of education? Thus, a meta-analysis was conducted to address these issues.

### 1.1 Conceptual definitions

#### 1.1.1 Subjective well-being

The exploration of well-being can be dated back to the ancient Greek period when Socrates expressed “what is the best way to live,” leading scholars to explore the concept of well-being. The early understanding of well-being was mainly based on Aristotle’s “perfectionism” and Jeremy Bentham’s “happiness,” which focused on literary definition. Since the 1950s, scholars from different fields such as psychology, sociology, and economics have begun defining and quantifying SWB in their respective ways. Psychological literature can be divided into two approaches. The first approach emphasizes how a person evaluates his own life, both emotionally and cognitively. According to Diener (1984) and Tov (2018), SWB refers to a person’s overall assessment of how satisfying his life is and whether he gets the things he wants in life. It often consists of frequent pleasant feelings, infrequent unpleasant feelings, and a holistic evaluation of life satisfaction. Thus, based on a person’s own life situation and some criteria they set; they possess the SWB when they have a comprehensive judgment of their life. The second approach is dedicated to pursuing activities that are consistent with values and goals. Ryff and Keyes (1995) believe that well-functioning individuals should be able to independently express themselves within their own internal standards, establish harmonious relationships with others, actively accept everything about themselves, have confidence in facing challenges in life and improving themselves, have clear and firm goals and objectives for life, have the ability to manage stress, and seize important opportunities. In the sociology literature, SWB refers to whether people feel good depending on social comparison with variable standards such as social equality or social cohesion, and not an individual-level concept but about collectives (Veenhoven, 2008). From the perspective of economics, especially in the happiness economics literature, SWB is viewed as the sum of good and bad feelings, which are important outcome measures, including employment, education, and health care (Easterlin, 2004). As for the components of SWB, economists, psychologists, and sociologists have conducted a lot of research and have found many distinct characteristics, but not entirely independent; they do overlap, such as real-time assessments of life experiences, emotional state, purpose, or suffering in life.

Although there are various expressions of SWB from different perspectives, we focus on the definition of SWB as a person’s subjective evaluation on of his own life quality as a whole (Diener, 1984; Diener et al., 1999). SWB encompasses frequent positive or pleasant emotions, rare negative or unpleasant emotions, as well as cognitive assessments such as life satisfaction (Tov et al., 2022). To ensure theoretical consistency, the indicators covered in this meta-analysis have been mapped to these conceptual frameworks. Life satisfaction measures and global happiness indicators are in keeping with the hedonic tradition (Waterman, 1993), whereas multidimensional scales such as the CASP-19, which assess control, autonomy, self-realization, and pleasure, are in line with Ryff and Keyes' (1995) and Diener (1984)‘s model. A number of approaches to measuring SWB have been developed, with self-reported judgments of overall life satisfaction or fulfillment being the most common (Wang et al., 2023). An example of a question proposed to measure SWB, originating from the World Values Survey, is “All things considered, how satisfied are you with life as a whole these days?” (Stone and Mackie, 2013). Alternative self-reported measures of SWB exist, such as a the CASP-19, a quality-of-life scale designed for older people (Hyde et al., 2003), a five-item scale was developed to measure cognitive judgments of life satisfaction from a global perspective.

#### 1.1.2 Education

There are several classifications of education; among these the most common definition merely refers to the level of formal education attained from primary, secondary, and tertiary education leading to certifications, such as diplomas and degrees. Another explanation defines education more broadly as formal and informal education. Informal education refers to learning through courses that do not provide diplomas and degrees and learning from news, social interaction, works of art and culture, training, and experiences related to work and life. For the purpose of this meta-analysis, we conceptualize education broadly, encompassing both formal and informal learning pathways that contribute to human capital development. More precisely, education is the process of learning different types of knowledge, opinions, or beliefs under a wide variety of circumstances; consequently, there are various teachers and teaching methods. However, recognizing the need for operationalization in empirical synthesis, we focus on measurable dimensions frequently studied in relation to SWB:

1. Educational level: The highest level of formal education completed (e.g., primary, secondary, tertiary degrees/diplomas) (Ardila et al., 2005).
2. Higher education participation: Students’ involvement in tertiary education programs (Chowdry et al., 2013).
3. Lifelong learning engagement: Participating in organized learning activities such as workshops, vocational training, non-degree courses, and self-directed learning related to professional or personal growth after finishing formal schooling (Hus, 2011).

This operationalization enables us to capture a variety of educational experiences by identifying specific characteristics. Our inclusion criteria give priority to research with quantitative results, even if we acknowledge broader definitions that include informal learning from social interactions and life experiences.

### 1.2 The relationship between education and SWB

In recent years, a large number of studies have examined the relationship between SWB and education, some of which believe that education has a direct impact on SWB (i.e., the development of cognitive abilities), while others find an indirect link between them (i.e., occupations, incomes, health, and social status). In terms of direct function, according to Stutzer (2004), middle-level education leads to the highest SWB. Tan et al. (2020) proposed a mechanism to prove that education can change one’s cognitive abilities and improve happiness and health. Moussa and Ali (2022) used simple linear regression and t-tests to reveal that students’ happiness levels correlated with their academic success during the COVID-19 lockdown period. Yu (2014) believes that public expenditure on education enhances SWB. Regarding indirect benefits on SWB, a study (Chen et al., 2020) indicated that education-occupation mismatch affects happiness; in particular, over-education positively affects happiness, whereas under-education has a minimal effect. A study (Yang et al., 2022) believed that the impact of education on individuals’ happiness could be mediated by income as an intermediary mechanism. Additionally, education can improve social status and happiness. Personal social status often comprises income, occupational status, and political status. Education, especially college education, can always be a priority for a party’s membership (Hu and Gao, 2019; FitzRoy and Nolan, 2020).

Scholars have not reached a consensus on how education affects subjective well-being. Although it is widely believed that education plays a crucial role in enhancing human capital and social well-being, empirical research on the relationship between education and well-being has shown contradictory results. This difference emphasizes the need for a thorough review of existing research to investigate how education directly and indirectly affects subjective well-being. Recent meta-analyses have mainly focused on individual-level correlations, ignoring structural moderating factors such as policy environments or urban–rural differences (Leite et al., 2024). Furthermore, psychological aspects such as self-compassion and the purpose of life are often overlooked. By combining the analysis of regulatory factors with an in-depth understanding of subjective well-being, this study aims to narrow these gaps.

Building upon this foundation, our research expanded this survey line through three key innovations: first, we conducted a more detailed examination of the specific dimensions of education, including the level of education, higher education participation and lifelong learning engagement, as well as their differential impact on subjective well-being. This fine-grained approach enables us to determine which aspects of education may have the greatest impact on SWB results. Secondly, we include regulatory factors, such as urban–rural differences, which may help explain the impact of the level of analysis. Third, our research provides several methodological advances: 1. we updated the previous meta-analysis and included all relevant publications from 2012 to 2024; 2. The time trend of the relationship between education and subjective well-being was evaluated by cumulative meta-analysis; 3. We systematically evaluated the direct and indirect mechanisms by which education promotes SWB achievement.

## 2 Methods

Meta-analysis, also known as quantitative research synthesis, is widely used in fields such as psychology and education. It can make more precise estimates of various results of the same issue in a single study and explain the heterogeneity of the results found in different studies. This meta-analysis followed the preferred reporting items of the guidelines for systematic Reviews and Meta-analyses (PRISMA 2020) (Page et al., 2021), and used the random-effects model (REM) to explain the expected heterogeneity of the studies. Based on the PICO framework, the core research questions of this study are as follows: Among adolescents and adults aged 12 and above, does exposure to differences in educational achievements (including different educational levels, participation in higher education, and engagement in lifelong learning) affect their subjective well-being (including core dimensions such as life satisfaction and emotional balance), and whether there are significant differences among groups with different educational levels (Cumpston et al., 2019).

We extended the previous systematic review and meta-analysis in four main ways. First, it covers a larger number of countries than previous studies. Not only are several developed countries (e.g., the UK and the US) collected in empirical evidence, but new countries (e.g., China, Korea, and Ecuador) are also added—for instance, in terms of the effect size (71.1%, in our study 39%). Thus, our results are more reliable. Second, our current meta-analysis adds to previous meta-analyses by including studies examining the impact of SWB on all possible forms of education. This is essential because, in the information era, individuals make progress whenever and wherever they are and ought to develop lifelong learning abilities. Third, although some relevant mediation variables have been noted in the literature (e.g., income, social status, and health), the present study adds several new ones, including data type and research design (Khan, 2022).

### 2.1 Eligibility criteria and exclusion criteria

For the purpose of this study, a set of eligibility criteria was defined that guided the selection of the literature. Specifically, the following three eligibility criteria were used: 1. The study should quantitatively examine the effect of formal or informal education on SWB. This means that the data included in this study were collected from any form of education, and some qualitative studies were excluded. 2. The study should be based on real data. 3. The study should report sufficient information to compute the effect size and its standard error (*t*-statistics, *p*-value, etc.). 5. Only one duplicate publication of the same data should be retained, for example, when both a journal article and a dissertation use the same data, select one as the primary source. The inclusion criteria for this study exclude non-empirical designs such as theoretical papers, reviews, and editorials, as well as studies focusing on irrelevant populations-specifically clinical groups (e.g., individuals with depression diagnoses) or children under 12 years of age. Additionally, studies with incomplete data (e.g., missing effect sizes or irreproducible results) are excluded, and for duplicate datasets, only the most comprehensive publication is retained.

### 2.2 Search strategy

**Figure 1:**
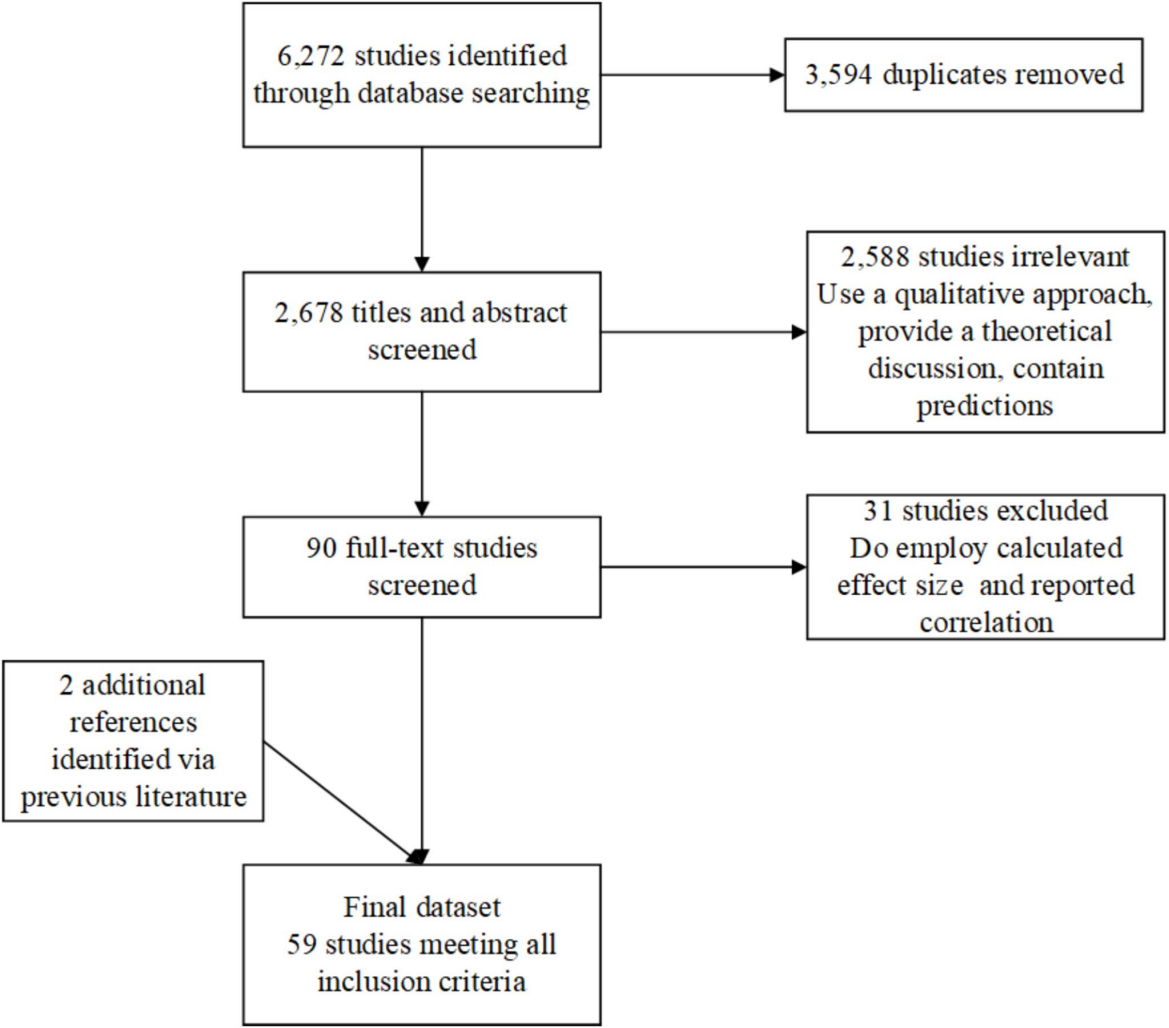
Flow chart of the search and screening process. Study-level inclusion criteria such as publication timeframe (2012–2023), language (English/Chinese), population age (≥12 years), and empirical design restrictions are detailed in the Methods section (“Search Strategy”).

We search comprehensively in seven electronic databases—Web of Science, Scopus, ScienceDirect, SpringerLink, ProQuest, Elsevier, and Google Scholar, to obtain relevant articles. The last search completed on September 6, 2023. Both “subjective well-being” or “happiness” or “life satisfaction” and “education” or “educational attainment” or “lifelong learning” were selected as search terms. To ensure that the results were relevant and manageable, we used the following filters: The study design was quantitative empirical (qualitative, theoretical, and predictive modeling papers were not included); the document type was limited to peer-reviewed journal articles; the publication years were 2012–2023; (1) Language: Chinese or English; (2) Population: 12 years of age and older. This process initially returned 6,272 records. After removing 3,594 duplicates and irrelevant studies, 2,678 titles and abstracts were screened. A further 2,588 were excluded based on eligibility criteria (e.g., qualitative design, theoretical focus). Burgess (2016) believes that education could be the key to growth and prosperity through the improvement of human capital; thus, growth and prosperity lead to an individual’s high SWB. The remaining 90 full-text articles were assessed in detail. We examined and extracted basic feature information and effect sizes of the findings from 61 studies and two studies were excluded at the final stage due to missing effect size information or incompatible statistical reporting formats. Finally, 59 were retained for meta-analysis. Reference lists of prior systematic reviews were also checked, yielding two additional studies. Figure 1 presents a PRISMA flowchart summarizing the search and selection process.

### 2.3 Theoretical basis and framework

**Figure 2:**
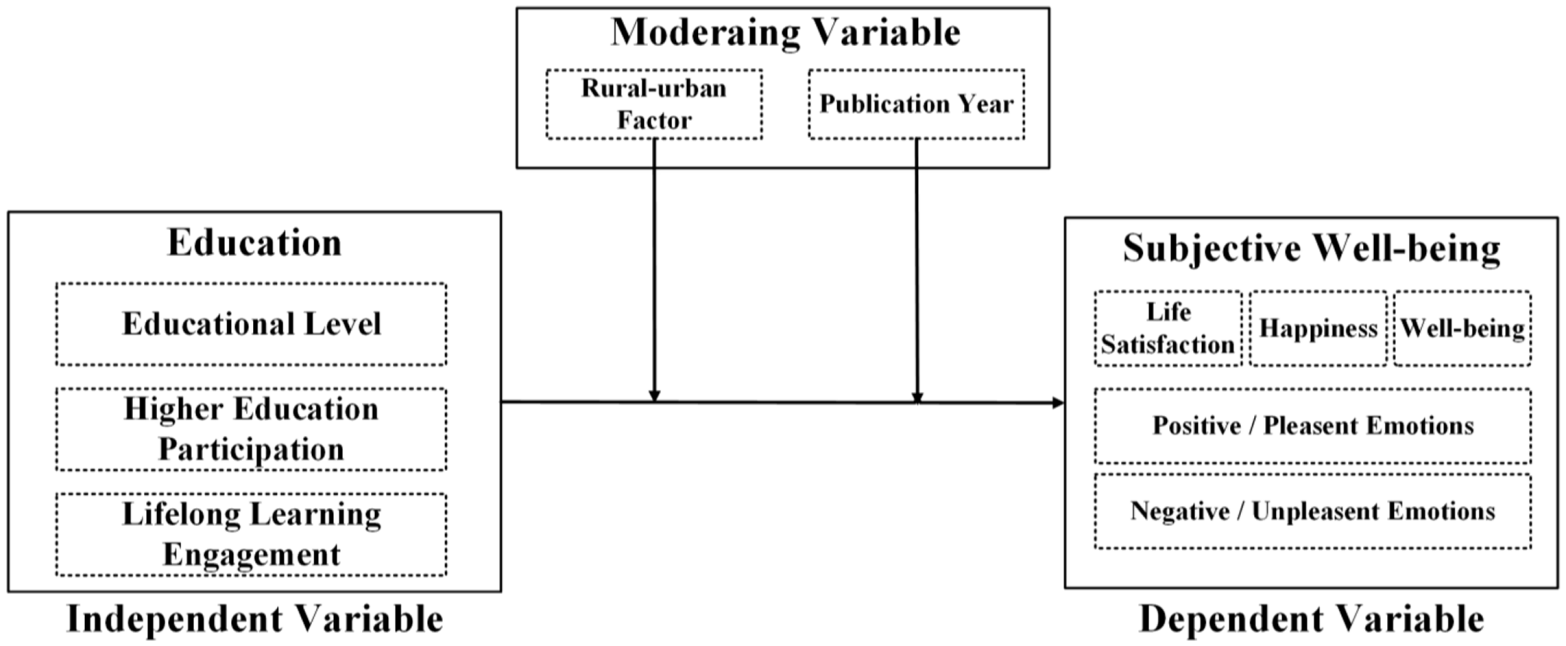
The conceptual framework of meta-analysis.

We have combined several theoretical stances from economics, sociology, and psychology to explain the connection between education and SWB. We have also offered theoretical justification for the moderating variables we chose. Social comparison theory and human capital theory are two main theories. According to the human capital theory (Becker, 1964; Schultz, 1961), education can improve a person’s productivity, skills and knowledge, which will improve their economic achievements (including income and employment) and non-monetary advantages. Because education provides people with the means to obtain life happiness, this hypothesis is consistent with our research results. The research results show that there is a strong positive correlation between education level and higher education participation and subjective well-being. Festinger's (1954) theory of social comparison indicates that individuals assess their own sense of happiness in relation to others. The gap between urban and rural areas in terms of educational opportunities (such as infrastructure and employment opportunities) has created different reference points, regulating SWB (Requena, 2016). In addition, we use the year of publication as a moderator to capture the potential time trend effect of the relationship between education and subjective well-being. In recent decades, educational opportunities, digital learning opportunities and social expectations for education have undergone profound changes, all of which may affect how education translates into subjective well-being (Kristoffersen, 2018; Leite et al., 2024). Including the year of publication helps to test whether recent studies systematically report different effects compared with earlier studies within our time frame. Figure 2 shows the conceptual framework of this study. It explains how to assume that three educational dimensions (educational level, higher education participation, and lifelong learning participation) influence SWB, and how urban–rural residence and publication years moderate these relationships.

### 2.4 Study coding

First, we investigated basic feature information comprised the title of the literature, lead author, publication time, journal, research object, sample size. Second, we recorded variable information and effect value information for each literature, such as variables name and correlation coefficient. To ensure conceptual clarity and consistency across studies, we adopted a broad definition of subjective well-being (SWB) following Diener (1984), which includes both cognitive components (e.g., life satisfaction) and affective components (e.g., happiness, emotional balance, positive and negative affect). While the original studies used varying terminology, such as “life satisfaction,” “happiness,” “subjective happiness,” or “positive emotions,” we reviewed and coded these outcomes based on their operational definitions in each study. Each of these variables was initially encoded separately to maintain the rigor of the analysis. However, according to previous meta-analyses (e.g., Diener et al., 2017; Gao et al., 2022), we then classified these variables under a broader subjective well-being structure for synthesis, as they reflect relevant aspects of mental health. This method can more comprehensively assess the relationship between education and overall subjective well-being. To be more specific, dependent variables mainly included SWB, and other kinds of variables which were related to SWB and designed as dependent variables in the extracted literature, such as life satisfaction, happiness, well-being, positive or pleasant emotions and negative or unpleasant emotions could also be involved in. Independent variables could be divided into two categories, one at macro level included education level (years of education, average education), higher education participation and the other at micro level included lifelong learning (Leite et al., 2024). And the rural–urban factor and publication year were the moderating variables. For the process of study coding, two researchers independently encoded the results according to the above coding rules, and the consistency of the results was 95.3%. After comparing the original texts, we negotiated and reached a consensus on the inconsistencies. Multiple independent studies from the same study were coded separately. Finally, we obtained 23,91,533 samples and 185 effect sizes. The basic feature encoding data (partial) are listed in Table 1.

**Table 1:** Basic feature coding data (partial).

Lead author (Year)	Literature source	Research object	Sample size	Effect size	Continent	Cut-off
Li et al. (2023)	Journal	Mixed population	6,133	Regression coefficient	Asia	≥5
Yue et al. (2021)	Journal	Urban residents	2,881	Regression coefficient	Asia	≥5
Zhao and Dai (2023)	Journal	Mixed population	12,498	Path coefficient	Asia	≥6
Sulaiman (2019)	Thesis	Mixed population	15,411	Regression coefficient	Asia	≥5
Ruiu and Ruiu (2019)	Journal	Mixed population	24,000	Regression coefficient	Europe	≥6
Kristoffersen (2018)	Journal	Mixed population	17,512	Regression coefficient	Australia	≥5

The “Cut-off” column refers to the minimum age threshold used to define the target sample population in each study. For example, “≥5” indicates that the study included participants aged 5 years or older.

Among the 59 original studies included, there were variable naming differences owing to different authors, research subjects, or translation reasons. We defined the concepts of variables in the original literature and set relevant items in the survey to merge variable names with the same meaning but different names. For example, when searching literature, “education level” often refers to various opportunities and pathways available to individuals and typically correspond to the number of years a person spends in formal schooling. Consequently, studies including “years of education” or “educational level” will be classified as “education level.” After summarizing the information on effect values, we selected education level, higher education participation, and lifelong learning engagement as influencing factors with combined effect values as the main research topic, and eliminated factors with fewer occurrences, such as homework anxiety and higher education expenditure.

Furthermore, we coded several moderator variables, that is, the factors potentially influencing SWB. The moderating variables were as follows:

1. Rural–urban factors: Some studies have found that education has a significantly positive effect on urban residents’ SWB, for example, city life can provide citizens easy-going transport, more employment opportunities and entertainment, which are conducive to the improvement of life satisfaction, whereas, there is no significant effect on rural residents’ SWB (Hu, 2017). Others found that the impact of education on rural SWB is greater than that on urban SWB (Luo, 2006). Moreover, Requena (2016) pointed out that how rural–urban factors affect SWB depends on a country’s economic level; more specifically, rural areas in less developed countries do not have complete communication, infrastructure, or good public services, which leads to unhappiness in people’s lives (Pehlivan et al., 2022). However, urban and rural residents in developed countries have similar living standards, and even city life has more negative effects, such as noise, pollution, and higher crime rates, leading to a less happy state in urban SWB. This shows that it was essential to consider rural–urban factors in our study.
2. Publication year: To find out possible time-trend effects in the association between education and subjective well-being (SWB), we added the year of publication for the moderator analysis. We also limited the host analysis to newer options (2018–2023) to ensure an adequate sample size for multiple years and to enhance the consistency of the results, even if the entire corpus of the included papers covers the period from 2012 to 2023. This period coincides with the rapid development of educational access and digital learning initiatives, as well as the growing global concern for well-being, especially after the COVID-19 pandemic. These structural and cultural shifts may have influenced how education affects SWB, justifying the inclusion of publication year as a moderating variable. In line with prior meta-analytic practice (e.g., Steel et al., 2008), we grouped studies into publication-year categories to examine whether more recent studies report systematically stronger or weaker effects than earlier ones within this focused window.

### 2.5 Meta-analysis process

To perform our study, we adopted the Comprehensive Meta-Analysis software 3rd edition (CMA 3.3) for quantitative analysis.

#### 2.5.1 Effect size calculation

To aggregate various reported estimates in the selected studies and be consistent with previous relevant meta-analysis (Bergquist et al., 2022; Crivelli et al., 2022; Liu et al., 2023), *r* (regression coefficient) is used as the selection criterion, and *F* values, *t* values, and *χ*^2^values that can be converted into r-values are also accepted. In case no information were missed, we also accepted the original literature that does not provide *r*-value directly but *β*. According to the formula *r* = 0.98β + 0.05*λ*, we could convert *β* to *r*, where *λ* is an indicator variable. When *β* is negative, it is equal to 0; when *β* is non-negative, it is equal to 1 (Peterson and Brown, 2005). Similarly, other estimates reported in the original literature were converted into corresponding formulas.

#### 2.5.2 Estimators and models

Generally, two approaches are used in meta-analyses: 1. the fixed-effects model (FEM) and 2. the random-effects model (REM). However, these studies differed in their assumptions. The FEM assumes that all included studies have a common true effect size and that all differences in the observed effects can be ascribed to sampling errors within the study. In contrast, REM assumes that the true effect size is different in every study and that the difference between the observed and true effect sizes is due to sampling error. According to the principles of a meta-analysis, only data with good homogeneity can be combined. Therefore, it is necessary to test the results of multiple studies for heterogeneity to select an appropriate effect model based on the heterogeneity analysis results. When there is remarkable heterogeneity in the study, the random effects model is preferred for analysis; whereas if the heterogeneity of the study is low, the fixed effects model will be used. The heterogeneity test usually uses *Q* test and *I*^2^ test. The criterion of the *Q* test is generally set at 0.10, and when *p* < 0.10, there is always heterogeneity between studies. The formula for calculating *Q* statistics is as follows (Li et al., 2018):


$$Q=\sum_{i=1}^{n}{\left(\frac{\theta_{i}-\bar{\theta_{i}}}{{\mathrm{se}}_{i}}\right)}^{2}$$

where $\theta_{i}$ is the effect size of the ith study, $\bar{\theta_{i}}$ is the average effect size of all the studies, and ${\mathrm{se}}_{i}$ is the standard error of the ith study.

The *I*^2^ statistic reflects the proportion of heterogeneity in the total variation in the effect size (Li et al., 2018). The value of *I*^2^ is from 0 to 100. The larger the *I*^2^, the more remarkable the heterogeneity. When 0 < *I*^2^ < 40, there was a low level of heterogeneity; when 40 < *I*^2^ < 60, there was moderate heterogeneity; when 60 < *I*^2^ < 75, the heterogeneity was remarkable; and when 75 < *I*^2^ < 100, there was great heterogeneity. The formula for calculating *I*^2^ is as follows:


$$I^{2}=\frac{Q-(K-1)}{Q}\times 100\%$$

*Q* is the chi-square value of the heterogeneity test and *K* is the number of studies included in the meta-analysis.

## 3 Results

### 3.1 Publication bias test

**Figure 3:**
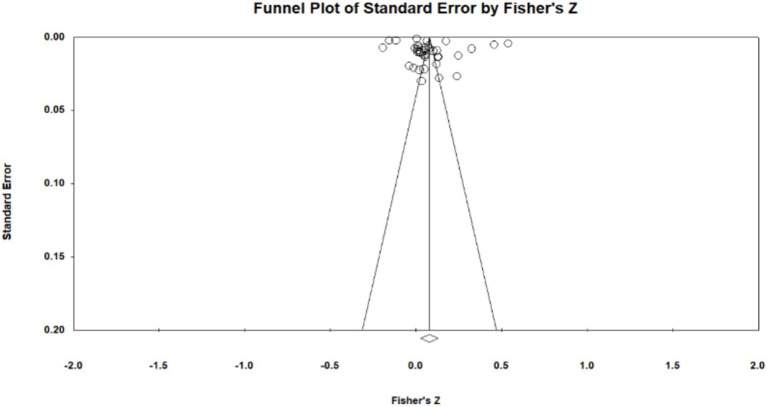
Funnel plot of relationship between education level and subjective well-being.

**Figure 4:**
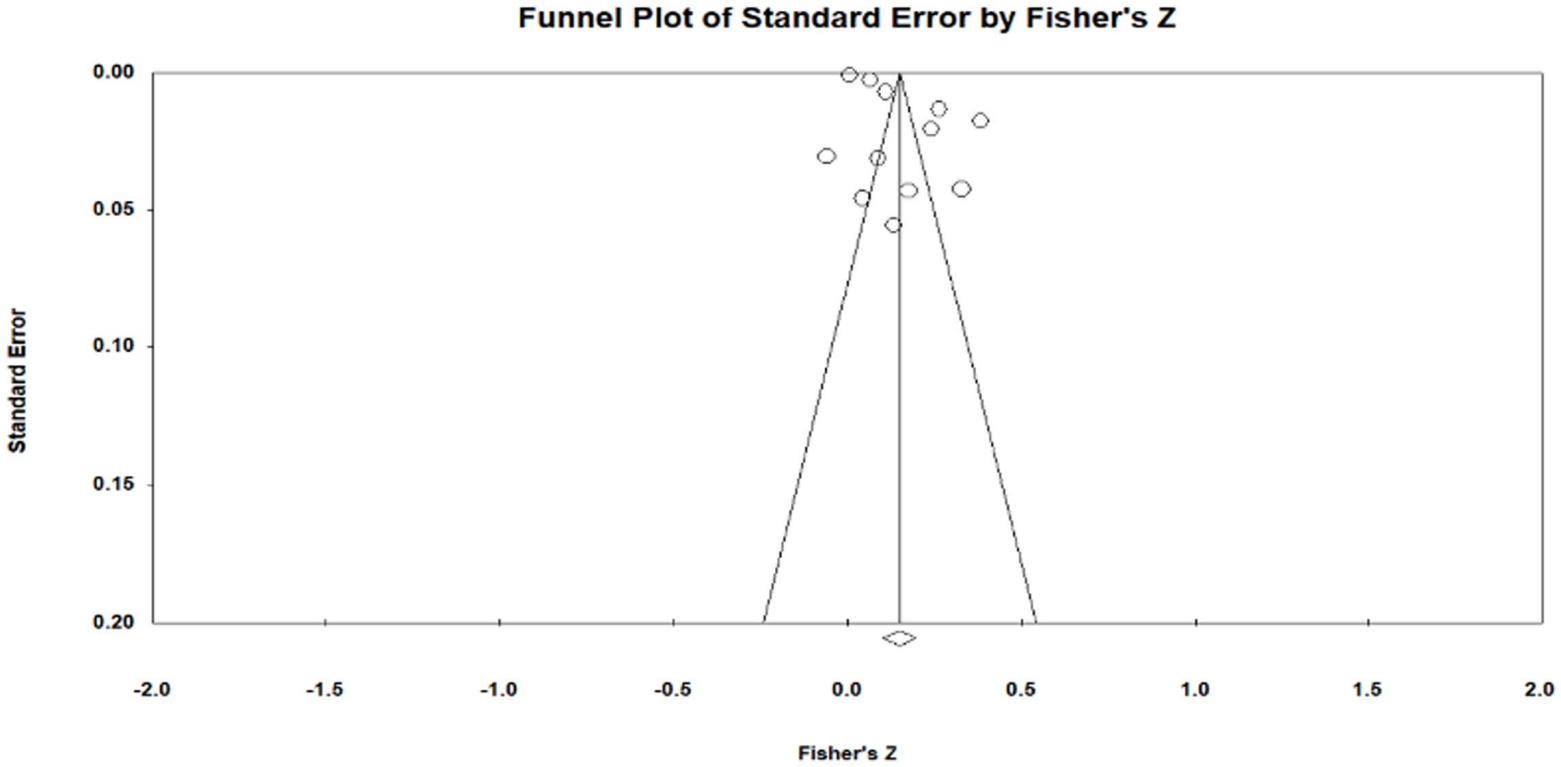
Funnel plot of relationship between higher education participation and subjective well-being.

**Figure 5:**
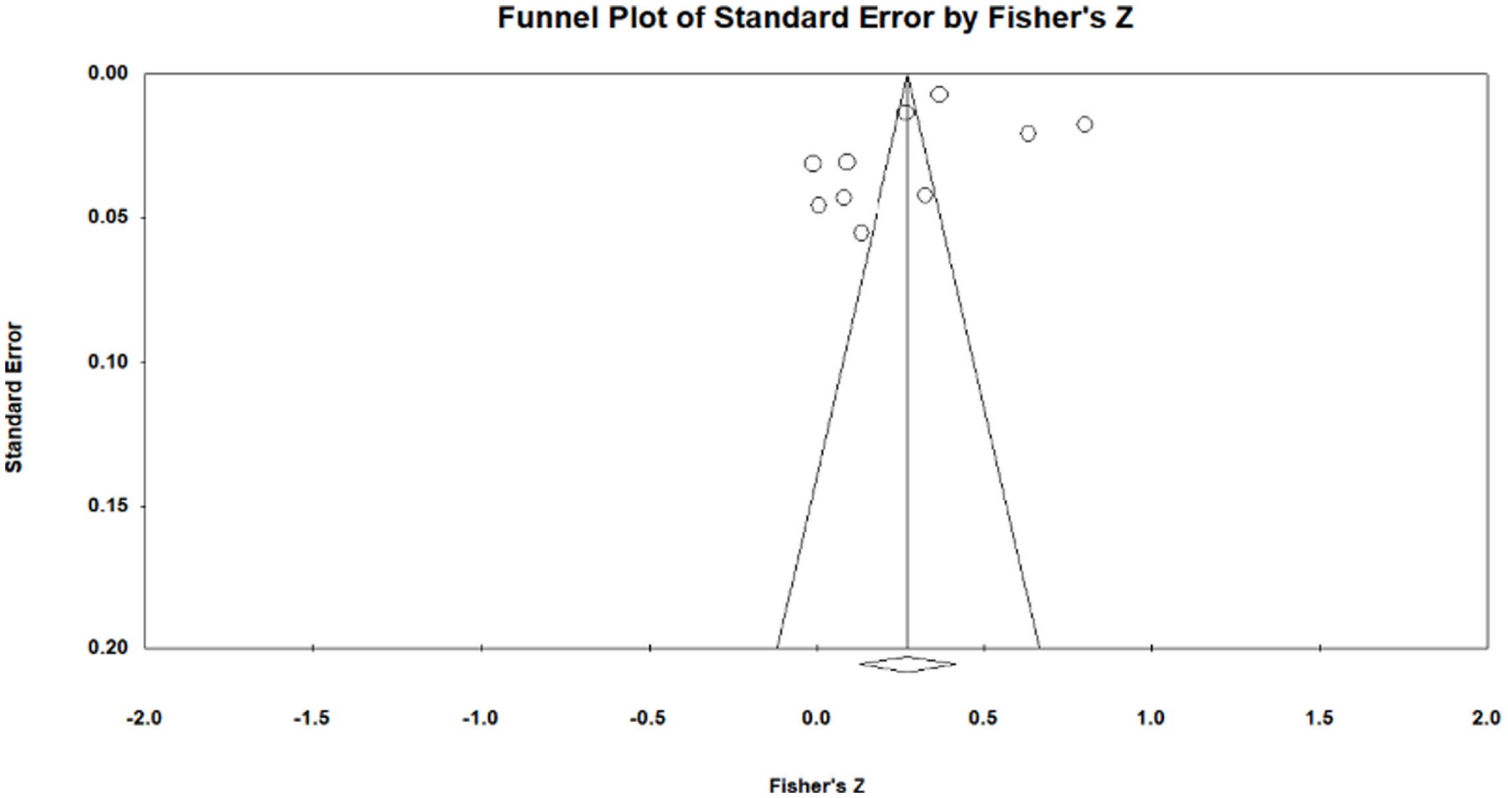
Funnel plot of relationship between lifelong learning engagement and subjective well-being.

Publication bias is the most effective way to systematically evaluate the validity of the results of a meta-analysis. The main reason for publication bias is incomplete retrieval owing to the poor quality of indexing or search strategy and insignificant study results. Moreover, articles whose research results are insignificant and sample sizes are small tend to be unpublished, leading to publication bias. To test for publication bias, we employed the qualitative funnel plot (Figures 3–5) and quantitative Egger and Rosentha tests. From the funnel plot, we found that both education level and higher education participation were evenly distributed around the effect size, whereas lifelong learning engagement was not. No significant publication bias was observed in the studies included in the meta-analysis.

The funnel plot is relatively intuitive, allowing researchers to visually determine whether there is bias in the study. However, different researchers may have different and inaccurate visual observations. Therefore, in addition to the funnel plot, we used the Egger and Rosenthal tests to determine whether there was publication bias. The data are presented in Table 2. For all of the influencing factors, on the one hand, in the Egger test, all the *p*-value are more than 0.05. On the other hand, all fail-safe numbers (Nfs) are greater than 5 K + 10, which is used to measure the impact size of publication bias; that is, the larger the Nfs, the weaker the impact of publication bias. From this, we can see that the literature included in our study did not have a publication bias. Visual inspection of funnel plots (Figures 3–5) showed approximate symmetry, suggesting a low risk of publication bias. Although the plots do not display pseudo-confidence regions, trim-and-fill analyses were performed and yielded no imputed studies. Egger’s regression tests for asymmetry were also non-significant (*p* > 0.05), supporting the robustness of the findings.

**Table 2:** Publication bias test results.

Influencing factors	K	*N*	Fail-safe N			Egger’s test		
			Nfs	*Z*-value	*p*-value	Egger’s intercept	SE	*p*
Education level	37	17,71,854	1,029	56.792	*p* < 0.001	9.640	6.789	0.164
Higher education participation	12	5,15,979	7,802	98.859	*p* < 0.001	−28.333	9.456	0.130
Lifelong learning engagement	10	30,299	2,948	46.268	*p* < 0.001	−7.753	2.844	0.433

*p < 0.05, **p < 0.01, ***p < 0.001. K = number of studies; *N* = total sample size; Nfs = fail-safe N; SE = standard error; Egger’s intercept = regression-based intercept used in publication bias test.

### 3.2 Heterogeneity

Sampling errors can lead to differences between the true and observed effect sizes in a practical study. Thus, it is necessary to perform a heterogeneity test to ensure that the random effects or fixed effects model can be used in the subsequent analyses. In our study, we used the *Q* and *I*^2^ tests to examine heterogeneity. When the *Q* test results are less than 0.05 and the value of *I*^2^ is greater than 75%, it can be considered a large heterogeneity (Higgins et al., 2003). Table 3 presents the results of the *Q* and *I*^2^ tests, which show that the results of each influencing factor’s *Q* test results are less than 0.05, and *I*^2^ test results are all greater than 75%. Therefore, there is great heterogeneity among the influencing factors, and we chose to use a random model for overall and moderating effect analyses to determine the sources of heterogeneity, except for sampling errors.

**Table 3:** Overall effect test and heterogeneity test results of each influencing factors.

Influencing factors	K	*N*	*r*	95% CI		Test of two-tailed		*Q*-value	df (Q)	*p*-value	*I* ^2^
				95% lower limit	95% upper limit	*Z*-value	*p*-value				
Education level	37	17,71,854	0.709	0.028	0.130	3.008	*p* < 0.01	33567.000	36	*p* < 0.001	99.893
Higher education participation	12	5,15,979	0.522	0.041	0.201	2.955	*p* < 0.01	3131.127	6	*p* < 0.001	99.808
Lifelong learning engagement	10	30,299	0.406	0.076	0.329	3.078	*p* < 0.01	528.250	6	*p* < 0.001	98.864

K represents the number of studies included in this study, *N* is the total sample size of K samples, *Q* is the heterogeneity value, df (Q) is the degree of freedom, *P*-value is used for heterogeneity testing significance value, *p < 0.05, **p < 0.01, ***p < 0.001, and *I*^²^ is the proportion of heterogeneity in the total variation in effect size.

In addition to statistical heterogeneity, conceptual heterogeneity may also arise due to differences in study design, evaluation instruments, and contextual circumstances. For instance, under some circumstances, having more education may even have the reverse effect on happiness (Araki, 2022). This paradox arises when the expansion of higher education raises people’s expectations of life, leading to more stress and dissatisfaction, especially in environments with lower economic returns or competitiveness. These findings help explain why different studies have different educational SWB relationships. Including such non-significant or negative data not only removes publication bias but also emphasizes the importance of moderator exploration and subgroup analysis in identifying the situations in which education may or may not enhance well-being.

### 3.3 Overall effect

Given that significant heterogeneity had been tested previously, a random-effects model (REM) was chosen for overall effect testing, and the statistical results are detailed in Table 3. According to Cohen (2013), if the *r*-value is between 0.50 and 1.00, it can be considered two variables as strong correlation; if the *r*-value is ranging from 0.30 to 0.49, there will be a moderate correlation; when the *r*-value is between 0.10 and 0.29, it indicates a weak correlation; and if the *r*-value is between 0.00 and 0.09, there is no correlation. Concurrently, if the *p*-value is less than 0.05, it also indicates the relationship of two variables is significant. From Table 4, we can see that education level and SWB have a strong relationship because the *r*-value is 0.709, which is larger than 0.5, and the *p*-value is 0.003, which is less than 0.05. Higher education participation was also strongly related to SWB, with an *r*-value of 0.522 and a *p*-value of 0.003. Lifelong learning engagement had a moderate relationship with SWB, with an *r*-value of 0.406 and a *p*-value of 0.002. Generally speaking, the three items mentioned above can be seen as important factors influencing SWB directly and significantly, which inspired us to accept education as much as possible within one’s ability to improve SWB.

**Table 4:** Test results of rural–urban factor as a moderating variable.

Independent variable	Moderator variable	Category	Qb	*k*	*r*	95% CI		*I* ^2^	Qw
						Lower	Upper		
Education level	Rural–urban factor	Rural	9.837***	8	0.131	0.088	0.121	79.953	41.577**
		Urban		11	0.238	0.187	0.286	92.885	498.724***
		Mixed		18	0.237	0.027	0.030	99.417	1963.000***
Higher education participation	Rural–urban factor	Rural	6.753**	3	0.256	0.253	0.259	86.819	181.876**
		Urban		4	0.182	0.166	0.182	96.511	240.265***
		Mixed		5	0.394	0.115	0.137	93.692	56.641**
Lifelong learning engagement	Rural–urban factor	Rural	4.341	2	0.206	0.029	0.148	48.445	479.000*
		Urban		3	0.435	0.094	0.669	98.938	539.000*
		Mixed		5	0.021	−0.377	−0.236	43.123	21.94*

Qb = between-group heterogeneity; Qw = within-group heterogeneity. *p < 0.05, **p < 0.01, ***p < 0.001.

### 3.4 Differences across education categories and heterogeneity interpretation

To further understand how different dimensions of education relate to SWB, we examined the effect three categories: years of education, higher education participation, and lifelong learning engagement. All three categories, years of education (*r* = 0.709, *p* < 0.05), higher education participation (*r* = 0.522, *p* < 0.05), and lifelong learning engagement (*r* = 0.406, *p* = 0.002), exhibited statistically significant positive associations with SWB, as indicated in Table 3. Although all effects were significant, their magnitudes differed. The highest correlation with SWB was years of schooling, which may reflect the cumulative advantages of extended exposure to education. The mild impact of higher education participation may be associated with socioeconomic advantages. The impact of participating in lifelong learning is relatively small but still significant, most likely due to age-related differences and self-selected participation.

Regarding heterogeneity, Table 3 shows the high *I*^2^ values (all >75%) for all categories, indicating substantial differences in effect size. This provides a basis for the application of random effects models. In addition, the observed changes exceeded predictions based solely on sampling errors, as indicated by a large number of *Q*-values (*p* < 0.05). This gap may be caused by differences in background factors such as research population, evaluation methods, national laws or behavioral norms. Although visual plots such as forest plots or meta-regression graphs are not included here due to space constraints, the numeric indicators reflect robust and differentiated effects across categories of education. Future studies could further unpack these patterns using graphical tools or explore more fine-grained educational distinctions.

### 3.5 Moderating effect

The results of the heterogeneity test showed that there is great heterogeneity among the included studies. To further explore the source of heterogeneity so that we can know the indirect impact of education on SWB, we operated a moderating effect. According to the literature included in our study, Cussianovich and Rojas (2014) believes that urban students tend to gain more education and are always provided with more skills and complete cognitive abilities, which can help them cope with the situation in which they live and then improve their own SWB. Jin et al. (2020) found that the positive effect of education was greater among urban residents than among rural residents. Therefore, we chose the rural–urban factor and typical moderator-publication year for the moderating effect test. The results of the moderating effects are presented in Tables 4, 5. From Table 4, we can see that rural–urban factors can significantly moderate the relationship between education and SWB; the significant moderating effect is greater in urban residents than in rural residents. More specifically, in the moderating role of rural–urban factors, the effect sizes of two influencing factors—education level (Qb = 9.837, *p* < 0.01) and higher education participation (Qb = 6.753, *p* < 0.01)—were all significantly different, whereas the difference in the effect size of lifelong learning engagement was weakly significant (Qb = 4.341, *p* < 0.05). In contrast, the moderating effect of publication year was overall not statistically significant across the five-year period (2018–2023). However, subgroup comparisons showed limited differences: for the “years of education” factor, studies published in 2020 yielded significantly higher effect sizes (Qb = 4.225, *p* < 0.001). For the “higher education participation” factor, significant variation was found across studies published in 2018, 2019, and 2023 (Qb = 5.089, *p* < 0.001). The “lifelong learning” factor exhibited weakly significant variation, with studies from 2019 and 2020 showing slightly elevated effect sizes (Qb = 3.236, *p* < 0.05). While these findings indicate some fluctuation, no consistent upward or downward trend was observed across years.

**Table 5:** Test results of the publication year as a moderating variable.

Independent variable	Moderator variable	Category	Qb	*k*	*r*	95% CI		*I* ^2^	Qw
						Lower	Upper		
Education level	The publication year	2018	4.225	2	−0.046	−0.321	0.236	99.825	46.70
		2019		4	0.120	−0.022	0.257	99.636	190.16
		2020		2	0.100	0.045	0.155	74.372	1263.768***
		2022		7	0.073	−0.010	0.156	99.518	548.80
		2023		6	0.223	0.029	0.401	99.913	97.907*
Higher education	The publication year	2018	5.089	3	0.256	0.253	0.259	85.154	355331.654***
		2019		2	−0.060	−0.083	−0.037	96.638	7498.981***
		2020		2	−0.060	−0.309	0.198	92.860	41506.00
		2022		2	0.172	−0.126	0.441	89.821	25104.33
		2023		3	0.061	0.038	0.084	73.237	7170.002***
Lifelong learning engagement	The publication year	2018	3.236	2	0.220	−0.043	0.455	96.328	161.61
		2019		2	0.374	0.094	0.599	96.379	3537.012*
		2020		2	0.423	0.094	0.669	99.540	7875.001*
		2022		2	0.189	−0.164	0.499	98.385	19778.45
		2023		2	0.030	−0.059	0.118	65.955	1565.64

Qb = between-group heterogeneity; Qw = within-group heterogeneity; *p* < 0.05 indicates statistical significance, *p < 0.05, **p < 0.01, ***p < 0.001.

## 4 Discussion

### 4.1 The relationship between education and SWB

#### 4.1.1 Education level

Educational level exerted a significantly positive influence on SWB (*r* = 0.709), which was the factor that had the most positive impact on SWB in our study. It can be attributed to the following reasons which are consistent with the results of several previous studies. On the one hand, education benefits people through the accumulation of knowledge capital; therefore, the more years of education one gets, the higher the level of one’s self-awareness will be, thus enhancing one’s SWB, which may be partially explained by the accumulation of cognitive skills, personal agency, and perceived self-efficacy developed through sustained educational engagement, rather than short-term rewards such as exam grades (Cheng et al., 2014). This is because a higher education level indicates that one has passed more tests, and both their level of self-recognition and intelligence have improved, resulting in a stronger sense of social identity and higher SWB. On the other hand, education increases the value of human capital through its impact on health and spiritual realms (Cheng et al., 2015). This means that those who accept education as much as possible accumulate rich knowledge covered by all aspects of education at different levels, such as psychology and health education. Consequently, he knew how to manage his life well and felt happy.

#### 4.1.2 Higher education participation

Higher education level was also significantly associated with SWB (*r* = 0.522). This can be linked with the characters of the higher education. For example, compared to other kinds of education, higher education participation tends to bring higher economic returns, safer and more stable jobs, and improved living conditions and may also support higher levels of social capital and life stability. Furthermore, a good higher education experience can effectively promote the development of one’s rational reflection on their connection with the objective world and form a rational concept of happiness that goes beyond simple hedonism (Long and Dai, 2019). Therefore, higher education is an effective way to achieve higher SWB.

#### 4.1.3 Lifelong learning engagement

Lifelong learning participation was positively correlated with subjective well-being, but weaker than expected (*r* = 0.406), indicating partial support for the hypothesized main effect. There are a few potential explanations for this. First, lifelong learning plays a crucial role in developing self-worth and a sense of achievement. Lifelong learning is a process of continuous learning throughout one’ life and allows individuals to acquire new knowledge and skills (Deci and Ryan, 2000). When a person becomes more professional and familiar with one specific field, they will feel a sense of satisfaction and achievement that promotes confidence and satisfaction. Second, continuous learning can bring new opportunities, expand one’s interpersonal network, make more like-minded friends and partners, and provide more possibilities for future personal development and achievement. Third, lifelong learning enhances adaptability. With the development of society and technological progress, working and living environments are constantly changing. Anyone may face difficulties and setbacks, and lifelong learning engagement can help individuals enhance their adaptability and meet challenges by continuously updating their knowledge and skills to adapt to changes in their work and living environment, allowing them to overcome difficulties better, improve their quality of life, and thus enhance their sense of happiness.

Based on individual level data from 59 studies, our research results indicate that participation in higher education (*r* = 0.522) and educational attainment (*r* = 0.709) significantly improve overall subjective well-being, while the impact of lifelong learning is relatively small (*r* = 0.406). This may be due to personal limitations in terms of time, resources, and motivation, as well as equal opportunities. Although our analysis is limited to micro-level correlations, macro mechanisms such as public education investment, social infrastructure, and institutional trust may further strengthen the relationship between education and SWB (Helliwell, 2003; Leite et al., 2024). In our meta-analysis, we regard these as potential effects rather than test effects. Well-educated societies generally appear to have higher levels of social cohesion, economic productivity, and institutional trust, which can enhance people’s well-being regardless of individuals’ educational backgrounds. In this way, education is a public interest that can improve social infrastructure and the quality of people’s lives. On the other hand, at the micro level, the acquisition of lifelong learning shows a relatively small impact size (*r* = 0.406). This might be due to the particularity of this kind of school education and the inequality in access to learning opportunities. Since not everyone has the time, resources or motivation to pursue lifelong learning, the impact of lifelong learning is limited.

Therefore, the government should not only promote individual participation in education, but also create an institutional environment in which education improves the economic, psychological and institutional structure of the community. Such macro-level effects reaffirm the importance of equitable, universal access to quality education as a strategy for improving national well-being.

### 4.2 The analysis of moderating effect

#### 4.2.1 Rural–urban factor

According to our test results, in the moderating effect, rural–urban factors significantly influenced SWB, especially in urban residents. Consistent with Hu (2017) and Jin et al. (2020), we found urban residents derive greater SWB benefits from education, likely due to better infrastructure and job markets. We believe that because of the industrialization of urban areas, only those who have gained higher education participation and strong skills are able to meet the needs of urban industrial transformation and economic development. Thus, this urban group can obtain more career opportunities, higher professional status, and better economic income, which will increase their SWB. Many low-quality workers with intermediate diplomas, especially junior high school diplomas, are gradually becoming marginalized, and their SWB is not much different from that of residents with primary school education or below because they are not equipped with the skills and abilities needed by the job market, which leads to low income and quality of life, as a result, they may have poor mental life but strive for surviving and feel low life satisfaction.

In addition to these structural advantages, psychological mechanisms may also help explain the urban–rural differences in how education influences SWB. According to the Social Comparison Theory, people in urban areas could have higher reference standards (such as friends who have earned university degrees), which increases the perceived positive effects of education on well-being. On the other hand, people in rural areas might compare themselves to members of smaller, more uniform groups. This could help to explain why there seems to be a larger correlation between education and SWB in metropolitan populations. However, we recognize that the concentration of lifelong learning programs and higher education institutions in cities may partially conceal the observed urban advantage. The divide between rural and urban areas is blurred since many people from rural areas move temporarily for school or to utilize online services. Furthermore, our research does not suggest that living in a rural area necessarily lowers well-being. Rural settings can promote high SWB through improved social bonds, reduced stress levels, and the advantages of a nature-based lifestyle, according to several research (e.g., Requena, 2016).

#### 4.2.2 The publication year

Surprisingly, during the five-year period (2018–2023), the moderating effect of the publication year was not statistically significant. Although individual differences were observed in some years, for instance, the effect values increased in studies published in 2020 or 2023, these fluctuations did not follow a consistent pattern. This might partly reflect a relatively short time frame, but it also indicates that the relationship between education and subjective well-being has remained stable over time. The concept of the stability of effect size has significant theoretical importance. Despite significant changes in the way education is provided during and after the COVID-19 pandemic (such as the increase in online learning), and the growing global focus on well-being, the overall association between education and subjective well-being (SWB) seems to remain consistent. This might indicate that the benefits for SWB brought by education will persist even when external conditions change.

In conclusion, through the integration of existing study results, we not only provide clarification and confirm the positive relationship between education and SWB, which supports research that emphasizes the role of education in SWB, but also demonstrate that rural–urban context significantly moderates the education-SWB link, highlighting the importance of addressing spatial disparities in educational access and outcomes.

These findings emphasize the importance of implementing key policies to promote educational equity. The government should give priority to increasing investment in rural education infrastructure, providing more reasonable lifelong learning projects for adults, and narrowing the gap between urban and rural communities in receiving higher education. For example, the establishment of online learning platforms in remote areas may help to narrow the gap in access to educational resources. The atmosphere and quality of schools affect students’ satisfaction, fairness and accessibility. A growing number of studies have shown that collaborative strategies, school culture reform, and social and emotional learning (SEL) are crucial to promoting students’ academic success and mental health (Ahmed, 2025). Combined with sel project, promoting positive interaction between teachers and students and creating an inclusive school environment will help to achieve structural changes aimed at improving students’ subjective well-being. These strategies are particularly important when expanding formal education alone may not be sufficient to ensure a significant increase in happiness.

Furthermore, these findings are particularly relevant for emerging countries, where the quality of education and socio-economic as well as regional differences are often very evident in terms of educational opportunities. In this case, promoting education equity not only needs to increase access to primary and secondary schools, but also needs to ensure that vulnerable groups have access to lifelong learning opportunities both economically and practically. For example, mobile learning platforms, subsidized community learning centers and localized adult education projects can help overcome obstacles in rural areas and underserved areas. Surprisingly, during the five-year period (2018–2023), the moderating effect of the publication year was not statistically significant. Although individual differences were observed in some years, for instance, the effect values increased in studies published in 2020 or 2023, these fluctuations did not follow a consistent pattern. This might partly reflect a relatively short time frame, but it also indicates that the relationship between education and subjective well-being has remained stable over time. The concept of the stability of effect size has significant theoretical importance. Despite significant changes in the way education is provided during and after the COVID-19 pandemic (such as the increase in online learning), and the growing global focus on well-being, the overall association between education and subjective well-being (SWB) seems to remain consistent. This might indicate that the benefits for SWB brought by education will persist even when external conditions change.

### 4.3 Limitations and practical implications

This study has various limitations. Firstly, the release window from 2018 to 2023 may not be long enough to capture the long-term changes in the relationship between SWB and education. Secondly, meta-analysis only includes papers published in English or Chinese, which may lead to language bias and limit the dissemination of research results in other language or cultural contexts. Thirdly, the relevance of the main studies included prevents any explicit causal reasoning between education and subjective well-being. Although significant associations were found, a third unmeasured variable, such as personality traits or baseline mental health, may affect the observed relationship. Despite these limitations, the findings are of great significance to policy makers and educators: promoting equitable access to quality education, whether formal or informal, is still a key way to support the well-being of the population. To sum up, these limitations provide several ways for future research: conducting longitudinal and cross lag group studies to assess causality; Integrate broader regulatory variables; Expand the coverage of language and culture to ensure the global applicability of research results.

## Data Availability

The original contributions presented in the study are included in the article/supplementary material, further inquiries can be directed to the corresponding authors.
